# Cytotoxic Effect of *Trypanosoma cruzi* Calcineurin B Against Melanoma and Adenocarcinoma Cells In Vitro

**DOI:** 10.1155/adpp/5394494

**Published:** 2024-11-28

**Authors:** Mayela Serrano-Rodríguez, Jorge E. Araya, Mauro Cortez, Patricio R. Orrego

**Affiliations:** ^1^Biomedical Department, Faculty of Health Sciences, University of Antofagasta, Antofagasta 1240000, Chile; ^2^Department of Medical Technology, Faculty of Health Sciences, University of Antofagasta, Antofagasta 1240000, Chile; ^3^Department of Parasitology, Institute of Biomedical Sciences, University of Sao Paulo, Sao Paulo 05508-000, Brazil

**Keywords:** antitumor protein, calcineurin B, *Trypanosoma cruzi*

## Abstract

Chagas disease caused by the obligate intracellular flagellate protozoan *Trypanozoma cruzi* infects about 6 million people. From the 1930s to the present, the antitumor capacity of *T. cruzi* has been studied; however, the identification of the responsible molecules for this effect remains undiscovered. Calcineurin, a calcium/calmodulin-dependent serine/threonine phosphatase, is a heterodimer consisting of a catalytic subunit (CaNA) and a regulatory subunit (CaNB). It has been described that *T. cruzi* CaN is involved in the cell invasion and proliferation of the parasite. Recently, extracellular human CaNB has been demonstrated to be capable of inhibiting tumor growth cells, conferring an antitumor effect; however, the extracellular role of *T. cruzi* CaNB (*Tc*CaNB) is still unknown. The objective of this work was to investigate the antitumor potential of *Tc*CaNB by interacting with membrane proteins and evaluating its effects on the viability, proliferation, and morphology of tumor cells in vitro. Additionally, the possible mechanism of action of *Tc*CaNB was explored. Murine melanoma (B16-F10), human cervical adenocarcinoma (HeLa), and African green monkey kidney epithelial (Vero) cell lines were employed for in vitro assays. Far Western blot and immunofluorescence were performed to assess the interaction of *Tc*CaNB with membrane proteins, and the effect of *Tc*CaNB on cell viability and proliferation was evaluated using the MTS assay and the CyQUANT NF assay, respectively. The effect of the caspase inhibitor Z-VAD-FMK on *Tc*CaNB-stimulated tumor cells was investigated to determine if *Tc*CaNB-induced cell death was associated with apoptosis. To assess cell cycle progression, *Tc*CaNB-treated cells were analyzed by flow cytometry. In this study, the results showed an interaction of *Tc*CaNB with cell membrane proteins in B16-F10 and HeLa tumor lines, indicating that *Tc*CaNB is capable of decreasing viability and proliferation of B16-F10 and HeLa cells, with no significant effect observed in Vero cells. Furthermore, morphological changes were observed in tumor cells treated with TcCaNB. DNA fragmentations and inhibition of caspases with Z-VAD-FMK partially counteracted the cytotoxic effects of *Tc*CaNB on tumor cells, suggesting that *Tc*CaNB-induced cell death might be associated with apoptosis. Additionally, *Tc*CaNB caused S phase cell cycle arrest in HeLa cells, with an increase in the sub-G1 population indicative of apoptosis, while no significant effects were observed in Vero cells.

## 1. Introduction

Calcineurin (CaN) is a ubiquitous serine/threonine protein phosphatase, which plays a fundamental role in Ca^2+^-mediated cellular responses associated with important physiological functions, including T-cell activation, cell cycle control, transcriptional regulation, apoptosis, among others [1]. CaN consists of a heterodimer formed by a catalytic subunit (CaNA) of 61 kDa and a regulatory subunit (CaNB) of 19 kDa [2]. CaNB contains four calcium binding EF-hand motifs, two of which have a low affinity and the other two have a high affinity for Ca^2+^ [3].

The role of CaNB is the regulation of CaN phosphatase activity, controlling the catalytic function of CaNA [4]; however, it has been shown that the role of human CaNB (*Hs*CaNB) not only regulates the phosphatase activity of CaNA, but cytosolic *Hs*CaNB is capable of interaction with proteins such as tubulin, heat shock protein 60 [5], procaspase-3 [6], and the Alpha type-7 subunit of the proteasome [7], conferring significant roles in the apoptosis and ubiquitin/proteasome pathways. On the other hand, extracellular *Hs*CaNB has been shown to modulate the immune system, inducing inflammatory cytokine production and responding as an effective adjuvant in cancer vaccine formulations [8–10], promoting macrophage proliferation by stimulating its phagocytic activity and enhancing cytokine secretion [11, 12], demonstrating its antitumor activity through interaction with these cells. Furthermore, *Hs*CaNB can inhibit the proliferation of gastric and hepatoma cancer cells through direct interaction with cancer cell lines [13].

CaN is present in different microorganisms, and the Ca^2+^-CaN signaling pathway is usually conserved in many eukaryotic pathogens [14]. In particular, in the human protozoan pathogen *Trypanosoma cruzi*, the etiologic agent of Chagas disease, there is evidence that critical processes for parasite life-cycle maintenance and gG can be mediated by calcium-dependent events through calcium-binding proteins, as well as by the presence of several kinases and phosphatases, which perform a fundamental role in the cellular signal regulation and integration [15]. In *T. cruzi*, there are two isoforms of the catalytic subunit *Tc*CaNA1 [16, 17] and *Tc*CaNA2 [18], which exhibit distinct cellular localizations (nucleus and cytoplasm, respectively), that play an essential role in parasite multiplication and host cell invasion [18]. On the other hand, the regulatory subunit of CaN in *T. cruzi* (*Tc*CaNB) possesses three EF-hand calcium-binding domains, with the first motif incomplete, displaying low calcium affinity, the second motif being complete, exhibiting high affinity, and the third motif being complete, showing low affinity in comparison to *Hs*CaNB [19]. The role of *Tc*CaNB is key in the invasion of parasite cells, and its marked difference on the primary structure scale with *Hs*CaNB makes it feasible to consider it as a potential chemotherapeutic target [17].

The inhibition of tumor cell growth in experimental mice models after infection with *T. cruzi*, first reported in the 1930s, is still attracting the attention of researchers today [20–26]. There is evidence that recombinant *T. cruzi* proteins possess antitumor activity, such as GP82, which induces apoptosis in melanoma cells [27]; P21, which is capable of abrogating the invasive phenotype of human breast cancer cells [28]; and calreticulin, which presents antiangiogenic activity and is capable of improving phagocytosis in macrophages [29–31].

The search for crucial *T. cruzi*-derived molecules with antitumor potential has gained importance and, therefore, interest in studying the role of *Tc*CaNB in the tumor microenvironment. The objective of this study is to evaluate in vitro the cytotoxic effects of recombinant TcCaNB in cancer cell lines.

## 2. Materials and Methods

### 2.1. Expression and Purification of the Recombinant *Tc*CaNB Protein

The full-length *T. cruzi* CaNB gen was previously cloned into a pGEX-1*λ*T expression vector [17]. The GST-*Tc*CaNB fusion protein was expressed in *Escherichia coli* BL21 (DE3) after addition of 1 mM IPTG and purified using glutathione-sepharose 4B (GE Healthcare, USA). Thrombin was used for GST-*Tc*CaNB protein cleavage (GE Healthcare, USA), and endotoxin was removed with Detoxi-Gel Kit (Thermo Scientific, USA). The purified *Tc*CaNB analysis was carried out by SDS-PAGE on 10% Coomassie blue stained gel and by Western blot analysis using mouse polyclonal anti-*Tc*CaNB antibody (provided by the Molecular Parasitology Laboratory, University of Antofagasta) and rabbit polyclonal anti-Human/mouse/rat Calcineurin B (anti-*h/m/r*CaNB) antibody (R&D Systems, USA). The contamination of endotoxin was quantified using the Pierce Chromogenic Endotoxin Quant Kit (Thermo Scientific, USA) and the contamination of LPS was 1 < EU/mL.

### 2.2. Cells and Cell Culture

Murine melanoma cells (B16-F10, ATCC, USA), human cervical adenocarcinoma cells (HeLa, ATCC, USA), and normal African Green Monkey kidney epithelial cells (Vero, ATCC, USA) were cultured in RPMI-1640 (Gibco, USA) supplemented with 10% fetal bovine serum (Gibco, USA) and antibiotic–antimycotic (Gibco, USA) at 37°C in a humidified incubator with 5% CO_2_.

### 2.3. Far-Western Blot (Far WB) Analysis

Far WB was performed as described by Wu, Li, and Chen [32] using the recombinant *Tc*CaNB and membrane proteins from the B16-F10, HeLa, and Vero cell lines. The extraction of membrane protein was done using the Mem-PER Plus Kit (Thermo Scientific, USA) and following the manufacturer's instructions. 100 μg of membrane proteins (prey proteins) and 10 μg BSA (negative control) were resolved in 10% SDS-PAGE and transferred to PVDF membrane, which were then incubated with decreasing concentrations of guanidine–HCl (6, 3, 1, 0.1, and 0 M) to denature and renature the prey proteins. The membrane was blocked with PBS 1X containing 0.05% Tween 20% and 5% skim milk and subsequently incubated with 10 μg *Tc*CaNB (bait protein). Mouse polyclonal Anti-*Tc*CaNB antibody (provided by the Molecular Parasitology Laboratory, University of Antofagasta) and rabbit polyclonal anti-*h/m/r*CaNB antibody (R&D Systems, EE. UU) were used as primary antibodies. Anti-mouse and anti-rabbit antibodies conjugated to horseradish peroxidase (Jackson ImmunoResearch, USA) were used to detect the membrane protein–*Tc*CaNB interaction. Immunocomplexes were revealed using a Clarity Western ECL Substrate (Bio-Rad, USA).

### 2.4. Immunofluorescence

Immunofluorescent analysis was performed according to standard techniques. 2 × 10^5^ B16-F10, HeLa, and Vero cells were seeded on glass coverslips in 24-well plates and grown overnight (ON) at 37°C with 5% CO_2_. After washing in PBS, the cells were incubated with 5 μg/mL *Tc*CaNB, 5 μg/mL BSA (negative control), and 10 μg/mL rat brain extract (BE) proteins (positive control) for 1 h at 37°C. Subsequently, the cells were washed in PBS and fixed with 4% paraformaldehyde in PBS for 15 min at room temperature (RT). The cells were then washed with PBS and blocked with PBS containing 2% BSA and 2% glycine for 1 h at RT. After washing in PBS, the cells were incubated ON at 4°C with rabbit polyclonal anti-*h/m/r*CaNB antibody diluted 1/50. The cells were washed and then incubated with Alexa Fluor 488 (Invitrogen, USA) for 1 h at RT and mounted with Fluoromount-G, with DAPI (Thermo Scientific, USA). Immunofluorescence images were taken with a *TC*S SP8 confocal microscopy (Leica Microsystems, Germany). Fluorescence intensity was measured with the Fiji image processing package (open source ImageJ software).

### 2.5. Cell Viability Assay

Cell viability was measured using the colorimetric MTS assay, CellTiter 96 Aqueous One Solution Cell Proliferation Assay (Promega, USA). 6 × 10^5^ B16-F10, HeLa, and Vero cells were seeded in 96-well plates and cultured ON at 37°C with 5% CO_2_ to achieve cell adhesion. Cells were treated with different concentrations of *Tc*CaNB (0, 2.5, 5, 10, 25, 50, 100 μg/mL) for 24 h at 37°C with 5% CO_2_. After treatment, 20 μL of MTS was added to each well and cells were incubated for 1 h at 37°C. Absorbance was measured at 490 nm using a microplate reader (Infinite M200 PRO, Tecan, Switzerland). Each concentration was replicated in 7 wells. Half-maximal inhibitory concentration (IC50) was determined as 50% decreased absorbance compared to the control group (0 μg/mL *Tc*CaNB). The data were expressed as percent cell viability compared to the control group. Furthermore, HeLa and Vero cells were incubated with different concentrations of *Tc*CaNB (0, 5, 10, 20 μg/mL) in the presence of Z-VAD-FMK ([20 μM]f, InvivoGen, USA), a cell-permeant pan caspase inhibitor of apoptosis. Each concentration was replicated in 7 wells. Data were expressed as percent cell viability compared to the group without inhibitor.

### 2.6. Cell Proliferation Assay

Parallel to the MTS assay, cell proliferation was confirmed using a CyQUANT NF Cell Proliferation Assay Kit (Invitrogen, USA), which is based on measurement of cellular DNA content via fluorescent dye binding. 6 × 10^5^ B16-F10, HeLa, and Vero cells were seeded in 96-well plates and cultured ON at 37°C with 5% CO_2_ to achieve cell adhesion. Cells were treated with different concentrations of *Tc*CaNB (0, 1, 5, 10, and 25 μg/mL) and for 24 h at 37°C with 5% CO_2_. After treatment, culture medium was removed and 100 μL of CyQuant working solution was added to each well and cells were incubated for 1 h at 37°C. The fluorescence of the sample was measured using a fluorescent microplate reader (Infinite M200 PRO, Tecan, Switzerland) at 485-nm excitation and 530-nm emission. Each concentration was replicated in 6 wells. Data were expressed as percentage of cell viability compared to the control group.

### 2.7. Morphological Observation

To explore whether *Tc*CaNB affects morphological characteristics in B16-F10, HeLa, and Vero cells, 6 × 10^5^ cells were plated into 96-well plates and cultured ON at 37°C with 5% CO_2_ to achieve cell adhesion. Cells were treated with increasing concentrations of *Tc*CaNB (0, 1, 5, 10, 25, and 50 μg/mL) for 24 h at 37°C with 5% CO_2._ Subsequently, the culture medium was removed and 100 μL of Tyrode's solution was added to each well. Observation of cell morphology was performed on ZOE fluorescent cell imager equipment (Bio-Rad, USA). Each concentration was evaluated by triplicated.

### 2.8. Detection of DNA Fragmentation by Agarose Gel Electrophoresis

1 × 10^6^ HeLa and Vero cells were seeded into 6-well plates and cultured ON at 37°C with 5% CO_2_ to achieve cell adhesion. Cells were incubated with 0, 5, 10, 20 μg/mL *Tc*CaNB and 100 μM H_2_O_2_ (positive control for apoptosis) for 24 h at 37°C. Cell DNA was released into lysis buffer (50 mM Tris–HCl pH 8.0, 62.5 mM EDTA, 2.5 M LiCl, 4% Triton X-100, 50 μg/mL RNase A) by rupturing the nucleus. Due to floating apoptosis cells, the culture medium was collected and centrifuged at 5000 rpm for 5 min, the supernatant was discarded and 500 μL of lysis buffer was added to the vacant plates, the lysate cells harvested from the plates were placed in the tubes that contained the centrifuged cell pellet, and incubated at RT for 5 min. The DNA in the supernatant was extracted using an equal volume of phenol:chloroform:isoamyl alcohol. The DNA was precipitated with ethanol, air dried, and dissolved in water quality molecular biology. The extracted DNA was quantified with an Infinite M200 PRO spectrophotometer (Tecan, Switzerland). DNA samples were electrophoresed on a 1% agarose gel containing 10 μL/100 mL SYBR-Safe DNA gel stain (Invitrogen, USA). The gel was examined and photographed by an imaging system ChemiDoc (Bio-Rad, USA).

### 2.9. Cell Cycle Analysis by Flow Cytometry

1 × 10^6^ HeLa and Vero cells were seeded into 6-well plates and cultured ON at 37°C with 5% CO_2_ to achieve cell adhesion. Subsequently, the culture medium was removed, and RPMI medium with 0.5% FBS was added for 24 h to synchronize the cells. After synchronization, the cells were incubated with different concentrations of *Tc*CaNB (0, 5, 10, and 20 μg/mL) in RPMI medium with 10% FBS for 24 h. After 24 h of treatment, the cells were collected by trypsinization. The harvested cells were washed with, centrifuged, and fixed with 70% ethanol for 20 min at 4°C. Once the cells were fixed, they were centrifuged, washed with PBS, and treated with 50 μg/mL RNAse A in PBS and incubated for 30 min at 37°C. Finally, the cells were stained with 2 μg/mL of propidium iodide (PI) in the dark for 15 min and analyzed by flow cytometry. Flow cytometry was performed using a FACSJazz (BD Biosciences). Data from the PI fluorescence were collected to a total count of 10.000 events. The cell cycle fractions G0/G1, S and G2/M were analyzed using the BD FACS software (BD Biosciences).

### 2.10. Statistical Analysis

Data are presented as mean ± standard deviation (SD). Statistical analysis was performed using the GraphPad Prism 8.0 software. Student's *t*-test was used to compare the fluorescence intensity of the immunofluorescence assay. One-way ANOVA and two-way ANOVA were used for multiple comparisons in cell viability and flow cytometry. A value of *p* < 0.05 was considered statistically significant.

## 3. Results

### 3.1. *Tc*CaNB Interacts With a Surface Membrane Protein of Melanoma and Adenocarcinoma Cells

**Figure 1:**
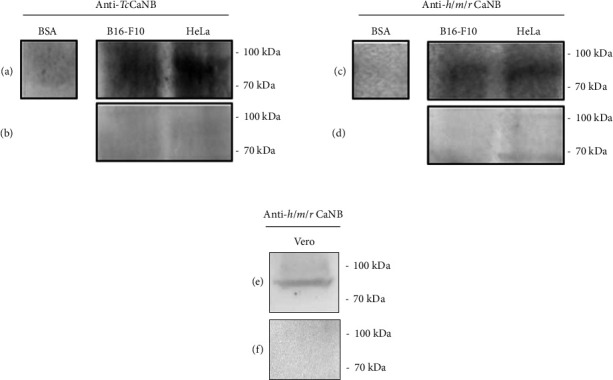
Far Western Blotting of recombinant *Tc*CaNB with membrane proteins from B16-F10, HeLa, and Vero cells. 100 μg of membrane proteins from B16-F10 and HeLa cells and 30 μg of recombinant *Tc*CaNB were used. The interaction was detected with anti-*Tc*CaNB and anti-*h/m/r* CANB antibodies. (a) Far WB using anti-*Tc*CaNB antibody for detection. (b) WB control using anti-*Tc*CaNB antibody. (c) Far WB using anti-*h/m/r* CaNB antibody. (d) WB control using anti-*h/m/r* CaNB antibody. (e) Far WB using anti-*h/m/r* CaNB antibody. (f) WB control using anti-*h/m/r* CaNB antibody. 10 μg of BSA was used as negative control in Far WB. 100 μg of B16-F10, HeLa, and Vero membrane proteins was used in WB controls.

To evaluate the interaction between *Tc*CaNB with B16-F10 and HeLa surface membrane protein, we first performed the Far Western blotting assay [32]. Membrane proteins were used as target, recombinant *Tc*CaNB as bait protein and BSA as negative target control, recognizing any protein–protein interaction using two types of antibodies (mouse polyclonal anti-*Tc*CaNB antibody and rabbit polyclonal anti-*h/m/r*CaNB antibody). The anti-*h/m/r*CaNB antibody was used, because it is also capable of recognizing *Tc*CaNB (Figure S1). Using the anti-*Tc*CaNB antibody, from the total of proteins, a band between 70 and 100 kDa was observed in both tumor cell samples (Figure 1(a)), a signal that it was not recognized using a BSA as control or the same antibody on a conventional Western blot (WB control) (Figure 1(b)). The same detected protein was observed when using an anti-*h/m/r*CaNB antibody (Figure 1(c)), without detection by WB control (Figure 1(d)), demonstrating a specific interaction between the *Tc*CaNB protein with the surface membrane protein of both B16-F10 and HeLa tumor cells. Furthermore, as a control of nontumor cells, a Far WB was performed with Vero cell membrane proteins, using the anti-*h/m/r*CaNB antibody. As shown in B16-F10 and HeLa cells, the results indicated the presence of a band between 70 and 100 kDa (Figure 1(e)), without detection by WB control (Figure 1(f)). The uncropped Far WB and WB control images can be found in Supporting Figure (Figure S2).

**Figure 2:**
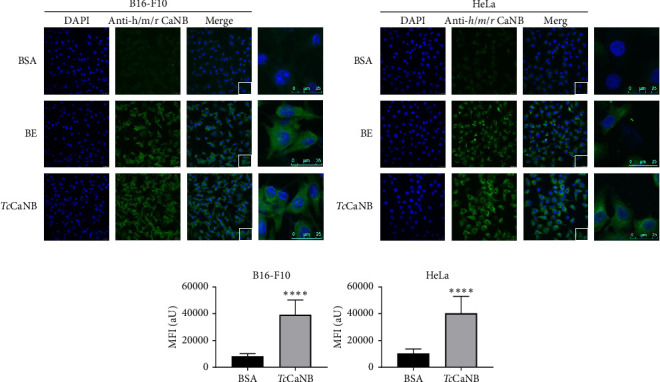
Interaction of the recombinant *Tc*CaNB protein with the cell surface proteins of B16-F10 and HeLa cells by an immunofluorescence assay. (a) B16-F10 and (b) HeLa cells were incubated with 5 μg/mL BSA (negative control), 10 μg/mL rat brain extract (BE), and 5 μg/mL *Tc*CaNB for 1 h at 37°C. Cells were visualized by confocal microscope after incubation with polyclonal rabbit IgG Human/Mouse/Rat Calcineurin B antibody followed by goat anti-Rabbit IgG Alexa Fluor 488 (green) and DAPI (blue). Scale bar: 25 μm. (c) Quantification of the mean fluorescence intensity under BSA (negative control) and *Tc*CaNB conditions. 100 cells were analyzed per condition. The results were expressed as average ± SD (Student's *t*-test, ⁣^∗∗∗∗^*p* < 0.0001).

Additionally, an immunofluorescence assay was performed using the anti-*h/m/r*CaNB antibody. Cells were incubated for 1 h at 37°C with BSA or rat BE proteins (BE) as negative and positive control, respectively, and *Tc*CaNB. The results show a strong fluorescence signal in B16-F10 (Figure 2(a)) and HeLa cells (Figure 2(b)) incubated with BE and *Tc*CaNB, which was confirmed by quantifying the intensities using an image processing program (Figure 2(c)), demonstrating that both rat-CaNB and *Tc*CaNB interact with the surface protein of tumor cells. The fluorescence signal from BE was not quantified because the amount of rat CaNB present in the BE proteins is variable and not comparable with the *Tc*CaNB concentration used for immunofluorescence.

### 3.2. *Tc*CaNB Affect the In Vitro Viability and Proliferation of Tumor Cells in a Dose-Dependent Manner

**Figure 3:**
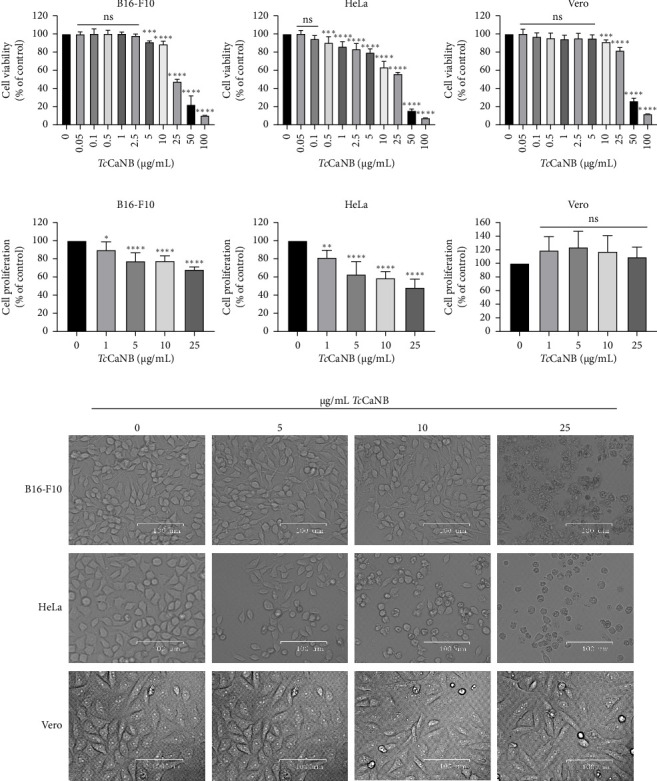
In vitro cytotoxicity of *Tc*CaNB in B16-F10, HeLa, and Vero cells. (a) Cell viability after 24 h of treatment with different concentrations of *Tc*CaNB in different tumor cells (B16-F10 and HeLa cells). Cell viability was determined by the MTS assay. Vero cells were included as normal cells. Results were expressed as average of triplicate ± SD (*n* = 7, one-way ANOVA and Dunnett's test, ⁣^∗∗^*p* < 0.01; ⁣^∗∗∗∗^*p* < 0.0001). Asterisks show statistical differences between *Tc*CaNB concentrations against control (0 μg/mL *Tc*CaNB). (b) Cell proliferation after 24 h of treatment with different concentrations of *Tc*CanB. Cell proliferation was evaluated with the CyQUANT kit. Results were expressed as average of triplicate ± SD (*n* = 7, one-way ANOVA and Dunnett's, ⁣^∗^*p* < 0.05; ⁣^∗∗^*p* < 0.01; ⁣^∗∗∗∗^*p* < 0.0001; ns: not significant). Asterisks show statistical differences for the different *Tc*CaNB concentrations with the control. (c) Cell morphology visualized by phase contrast of different cells tested, after 24 h of treatment with different concentrations of *Tc*CaNB. Scale bar, 100 μm.

The effect of *Tc*CaNB on cell survival was examined on B16-F10, HeLa, and Vero cells for 24 h. Since Vero cells are derived from normal tissue, the sample was included as a cell line control. First, IC50 was measured, obtaining 21.64 μg/mL (B16-F10 cells), 15.45 μg/mL (HeLa cells), and 34.14 μg/mL (Vero cells) (Figure S3). HeLa cells had the lowest IC50 value, and Vero cells the highest. Cell viability (measured by MTS assay) of the control group (0 μg/mL TcCaNB) was compared with treated cells with different concentrations of *Tc*CaNB (1, 2.5, 5, 10, 25, and 50 μg/mL *Tc*CaNB). Cell viability decreased as *Tc*CaNB concentrations increased, indicating the cytotoxic effect of *Tc*CaNB on tumor cells, affecting tumor cells more than Vero cells (Figure 3(a)). To evaluate cell proliferation, B16-F10, HeLa, and Vero cells were incubated with 0, 1, 5, 10, and 25 μg/mL *Tc*CaNB for 24 h and, after incubation, analyzed by CyQUANT kit. Cell proliferation decreased as *Tc*CaNB concentrations increased in B16-F10 and HeLa cells, showing no significant changes in Vero cells (Figure 3(b)). Cell morphology was recorded by cell imager equipment in the different sets of cells to complement the previous analysis. At increased concentrations of *Tc*CaNB, the morphology was affected only in tumor cells, showing no changes in Vero cells (Figure 3(c)).

### 3.3. The Cytotoxicity Effect of *Tc*CaNB in HeLa Cells Is Mediated by Apoptosis

**Figure 4:**
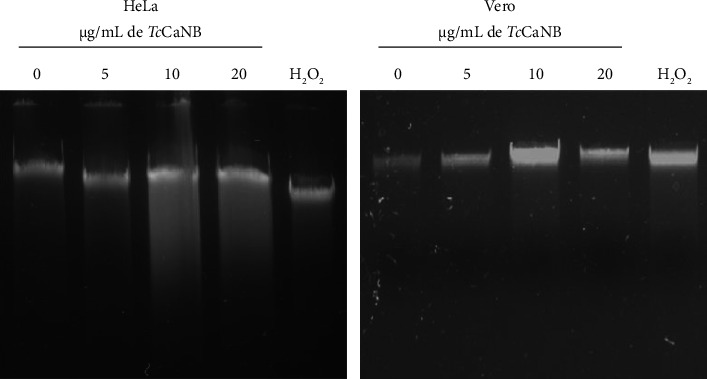
DNA fragmentation analysis by agarose gel electrophoresis in *Tc*CaNNB-stimulated tumor and nontumor cells. HeLa and Vero cells were treated with different concentrations of *Tc*CaNB and H_2_O_2_ (positive control of apoptosis) for 24 h. DNA visualization was performed using a 1% agarose gel.

To evaluate whether the cytotoxic effect of *Tc*CaNB is through apoptosis, we chose HeLa cells because they exhibit increased cytotoxicity in response to *Tc*CaNB. To elucidate whether *Tc*CaNB decreased cell survival by induction of DNA fragmentation (an important feature of cell apoptosis), genomic DNA was isolated from HeLa cells and Vero cells after exposure to different concentrations of *Tc*CaNB. H_2_O_2_ was used as a positive control of apoptosis. Results show that *Tc*CaNB treatment led to DNA fragmentation in HeLa cells in a dose-dependent manner in comparison with the intact DNA from untreated cells (Figure 4). As shown by the characteristic DNA fragmentation in agarose gels, apoptosis was induced at *Tc*CaNB concentrations of 10 and 20 μg/mL. Results show that, unlike HeLa cells, Vero cells did not exhibit significant DNA fragmentation under any of the tested conditions. There was no observable difference in DNA integrity across the various *Tc*CaNB concentrations (10 and 20 μg/mL) when compared to untreated controls (Figure 4).

**Figure 5:**
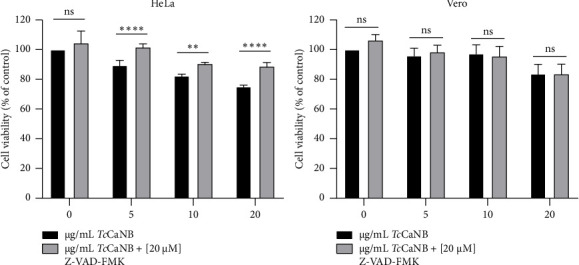
Effects of caspase inhibitor on cell viability in *Tc*CaNB-stimulated tumor and nontumor cells. HeLa and Vero cells were treated with different concentrations of *Tc*CaNB in the presence of Z-VAD-FMK ([20 μM]_f_) for 24 h. Cell viability was determined by the MTS assay. Results were expressed as average of triplicates ± SD (*n* = 4, two-way ANOVA and Sidak's test, ⁣^∗^*p* < 0.05; ⁣^∗∗∗^*p* < 0.001; ⁣^∗∗∗∗^*p* < 0.0001; ns: not significant). The asterisks indicate significant differences between the different *Tc*CaNB concentrations in the presence and absence of the Z-VAD-FMK inhibitor.

Furthermore, to confirm the involvement of caspases in *Tc*CaNB-induced apoptosis, the caspase inhibitor, Z-VAD-FMK was used [33]. HeLa and Vero cells were incubated with different concentrations of *Tc*CaNB in the presence of Z-VAD-FMK ([20 μM]f) for 24 h. Subsequently, cell viability was measured by the MTS assay. The results did not show changes in Vero cell viability with 5, 10, and 20 μg/mL *Tc*CaNB in the presence of Z-VAD-FMK compared to cells without inhibitor. However, cell viability increased in HeLa cells stimulated with 10, 20, and 40 μg/mL *Tc*CaNB in the presence of Z-VAD-FMK, compared to cells stimulated with only *Tc*CaNB (Figure 5).

### 3.4. *Tc*CaNB Induced S Phase Cell Cycle Arrest in HeLa Cells

**Figure 6:**
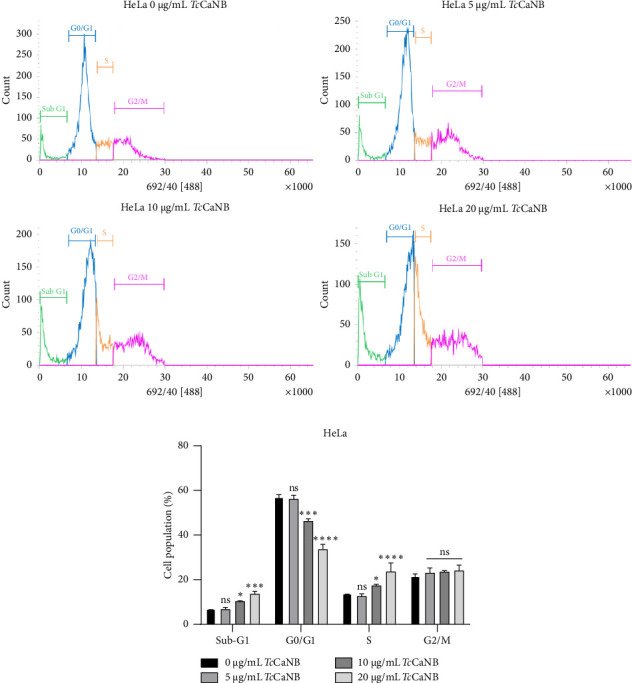
Cell cycle distribution assessed by flow cytometry in *Tc*CaNB-stimulated HeLa cells. (a) Cell cycle analysis of HeLa cells after 24 h of *Tc*CaNB treatment (0, 5, 10, and 20 μg/mL). (b) Quantification of the cell cycle analysis. Results were expressed as average of triplicates ± SD (two-way ANOVA and Dunnett's test, ⁣^∗^*p* < 0.05; ⁣^∗∗∗^*p* < 0.001; ⁣^∗∗∗∗^*p* < 0.0001; ns: not significant). Asterisks show statistical differences between *Tc*CaNB concentrations against control (0 μg/mL *Tc*CaNB) in each phase of the cell cycle.

**Figure 7:**
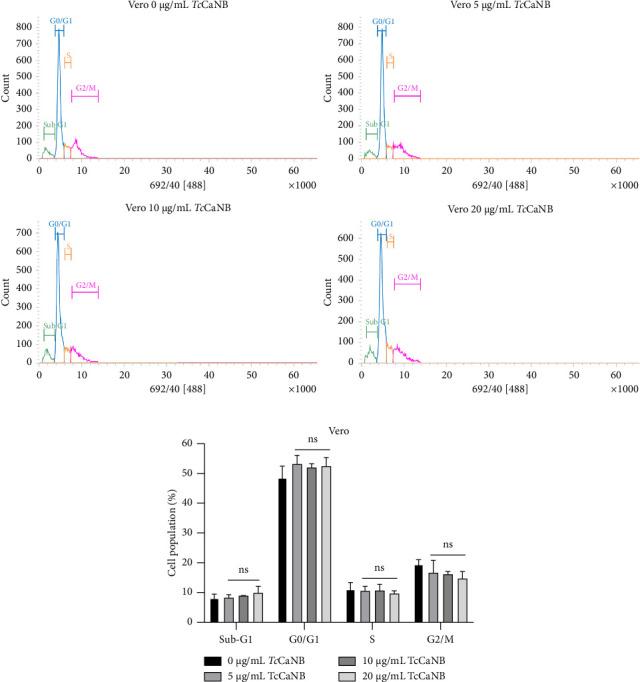
Cell cycle distribution assessed by flow cytometry in *Tc*CaNB-stimulated Vero cells. (a) Cell cycle analysis of HeLa cells after 24 h of *Tc*CaNB treatment (0, 5, 10, and 20 μg/mL). (b) Quantification of the cell cycle analysis. Results were expressed as average of triplicates ± SD (two-way ANOVA and Dunnett's test, ⁣^∗^*p* < 0.05; ⁣^∗∗∗^*p* < 0.001; ⁣^∗∗∗∗^*p* < 0.0001; ns: not significant). Asterisks show statistical differences between *Tc*CaNB concentrations against control (0 μg/mL *Tc*CaNB) in each phase of the cell cycle.

To investigate the cell cycle events underlying the observed cytotoxic effects of *Tc*CaNB, we evaluated the effect of different TcCaNB concentrations on cell cycle progression in HeLa and Vero cells. Cell cycle analysis was done using flow cytometry with PI to stain cellular DNA. According to the cell cycle analysis, the results showed that 10 and 20 μg/mL *Tc*CaNB inhibited the S phase of the cell cycle in HeLa cells. *Tc*CaNB increased S phase cell population in comparison to control cells, while reducing G0/G1 phase cell population. The results also showed a gradual increase in the sub-G1 population of treated HeLa cells, from 6.9% in the untreated control group to 10.4% and 13.7% when exposed to 10 and 20 μg/mL *Tc*CaNB, respectively, for 24 h (Figure 6 and Table 1). An increase in the percentage of cells in sub-G1 in a dose-dependent manner, suggesting the induction of apoptosis. Furthermore, no significant changes in the cell cycle were observed in Vero cells in the presence of *Tc*CaNB (Figure 7 and Table 2).

## 4. Discussion

Currently, the interaction of *Tc*CaNB with proteins present in human cells lacks documented information. Therefore, the initial phase of this investigation aimed to determine whether *Tc*CaNB could interact with any surface protein of tumor cells and consequently induce a cytotoxic effect. The results of the Far WB revealed the presence of a band, detected by anti-*Tc*CaNB and anti-h/m/r CaNB antibodies, demonstrating specific binding between *Tc*CaNB and a membrane protein with a molecular mass between 70 and 100 kDa, present in B16-F10 melanoma cells, HeLa adenocarcinoma cells and Vero kidney epithelial cells. This result was further confirmed by indirect immunofluorescence without cell permeabilization, revealing a surface adhesion pattern, providing the first evidence that *Tc*CaNB is capable of binding to a surface protein in melanoma and adenocarcinoma cells.

Currently, three receptors have been identified that interact with *Hs*CaNB: Integrin *α*M [34], CD14 [35], and TLR4 [36]. Although these receptors are found predominantly in immune system cells, such as macrophages [37–39], the presence and relevance of *α*M and TLR4 receptors in tumor cells have also been demonstrated [40, 41]. TLR4 is a transmembrane protein with an approximate mass of 95 kDa, identified as overexpressed in different types of cancer, playing key roles in the development and progression of this disease [41, 42]. Research on TLR4 has been conducted in skin and cervical cancer using B16-F10 melanoma and HeLa adenocarcinoma cell lines, respectively. The experimental evidence has shown significantly higher expression of TLR4 in B16-F10 melanoma cells compared to normal skin cells [43] and also demonstrate an increase in TLR4 expression in LPS-stimulated HeLa cells [44]. Moreover, it is also known that TLR4 is expressed in normal tissue, and it is widely recognized that in normal conditions, renal TLR4 expression is low; however, the expression of this molecule increases in response to renal injury and/or infection [45]. However, additional experiments are required to identify exactly the surface membrane protein with which *Tc*CaNB would be interacting.

On the other hand, it has been shown that *Tc*CaNB can play a key role in the cellular invasion of *T. cruzi* [17]. However, there are no reports on the extracellular function of *Tc*CaNB on human cells, independent of its regulatory properties on the phosphatase activity of *Tc*CaNA1 or *Tc*CaNA2. The extracellular function of *Hs*CaNB has previously been demonstrated, showing its capacity to inhibit cell proliferation in a variety of tumor models [8, 13, 46].

In this study, for the first time, it is shown that *Tc*CaNB has the ability to induce in vitro cytotoxicity in tumor models such as melanoma (B16-F10) and adenocarcinoma (HeLa). This cytotoxicity property significantly impacts on the viability, proliferation, and morphology of cancer cells. While in Vero kidney epithelial cells a decrease in cell viability is observed starting from 10 μg/mL *Tc*CaNB (IC50 = 34.14 μg/mL), but no effect is observed on cell proliferation or cell morphology. These results indicate that although *Tc*CaNB is capable of interacting with the three cell lines, the cytotoxic effect of *Tc*CaNB is more predominant in B16-F10 and HeLa cells (tumor lines), presenting low cytotoxicity in nontumor cells (Vero cells).

The cytotoxic effect of TcCaNB against tumor cells could be attributed to differences in expression of various membrane receptors. Integrin and Toll-like receptors (TLRs) can exhibit different expression patterns in tumor cells compared to normal cells, for example, in normal cells, CD11b (Integrin *α*M) is primarily expressed on myeloid cells and is involved in immune functions such as cell adhesion, migration, and phagocytosis. Its expression is tightly regulated and contributes to normal immune surveillance and responses [47]. However, in tumor cells and within the tumor microenvironment, CD11b expression can be upregulated, particularly on immune cells like tumor-associated macrophages (TAMs) and myeloid-derived suppressor cells (MDSCs). These cells can be co-opted by the tumor to promote an immunosuppressive environment, aiding in tumor progression, angiogenesis, and metastasis [41]. Otherwise, the expression of TLRs varies significantly between normal and tumor cells and their differential expression often correlates with disease prognosis [48]. In normal cells, TLRs primarily function as part of the innate immune system, detecting pathogens and initiating immune responses to protect the body. Their expression is generally regulated and balanced to prevent excessive inflammation [49]. In contrast, tumor cells often exhibit overexpression of specific TLRs, such as TLR2, TLR4, and TLR9. This overexpression contributes to cancer progression by promoting chronic inflammation, enhancing cell survival, and supporting immune evasion. For example, TLR4 overexpression in cancer cells can lead to resistance to apoptosis and increased tumor invasiveness [50, 51].

Although this study demonstrates the antitumor effect of *Tc*CaNB, the underlying mechanism supporting it has not been detailed. Regarding *Hs*CaNB, it has been shown that *Hs*CaNB overexpression can significantly increase TNF*α*-induced apoptosis by binding to mitochondria [52]. This is because exogenous CaNB can quickly enter cells through TLR4 receptors and generate cytotoxicity in some TLR4-rich tumor cells [36]. Apoptosis appears to be the main mechanism of *Hs*CaNB-induced cell death in hepatoma and gastric cancer cells, causing mitochondrial depolarization in an *Hs*CaNB-dependent manner, leading to the release of cytochrome c and the cleavage of the initiator caspase 9, a characteristic of numerous stimuli that cause apoptosis through the intrinsic pathway involving mitochondria [13, 46].

Based on the evidence presented by *Tc*CaNB in this study and comparing it with previously documented information on *Hs*CaNB, it could be inferred that the mechanism by which *Tc*CaNB induces the cytotoxic effect in melanoma and adenocarcinoma tumor cells is through apoptosis [33, 53]. The DNA fragmentation observed in HeLa cells, but not in Vero cells, indicates that *Tc*CaNB induces apoptosis in a dose-dependent manner. The DNA fragmentation observed in agarose gel electrophoresis is a hallmark of apoptosis, confirming that *Tc*CaNB triggers this programmed cell death process in HeLa cells. This result is consistent with the notion that TcCaNB may specifically target tumor cells, such as HeLa, which are more susceptible to its effects compared to normal cells like Vero cells. The absence of significant DNA fragmentation in Vero cells under all tested conditions suggests that TcCaNB does not induce apoptosis in these normal cells. This differential sensitivity could be attributed to differences in cellular mechanisms between HeLa and Vero cells, such as variations in drug uptake, intracellular signaling pathways, or apoptotic machinery [54, 55]. The lack of apoptosis in Vero cells highlights the potential selectivity of *Tc*CaNB toward cancerous cells, reducing the risk of collateral damage to normal tissues. The use of the pan-caspase inhibitor Z-VAD-FMK provides additional insight into the apoptotic pathway involved in *Tc*CaNB-induced cell death. The increase in cell viability in HeLa cells treated with *Tc*CaNB in the presence of Z-VAD-FMK, compared to cells treated with *Tc*CaNB alone, indicates that caspase activation is indeed involved in *Tc*CaNB-induced apoptosis. Caspases are key executors of apoptosis, and their inhibition partially protects HeLa cells from *Tc*CaNB-induced cell death. This result suggests that *Tc*CaNB's apoptotic effect is mediated, at least in part, through caspase-dependent pathways.

Regarding cell cycle analyses, the results provide insight into the mechanisms underlying the cytotoxic effects of *Tc*CaNB, particularly its impact on cell cycle progression and apoptosis induction. The observed inhibition of the S phase in HeLa cells treated with 10 and 20 μg/mL *Tc*CaNB indicates that *Tc*CaNB disrupts DNA synthesis and cell cycle progression. The increase in the S phase cell population alongside a reduction in the G0/G1 phase population suggests that *Tc*CaNB may impair the transition from the G1 phase to the S phase or hinder DNA replication. This S phase arrest can lead to the accumulation of cells with damaged or incomplete DNA, contributing to the observed cytotoxic effects. The significant increase in the sub-G1 population in a dose-dependent manner correlates with the induction of apoptosis. The sub-G1 fraction typically represents cells with fragmented DNA, a common feature of apoptosis. The rise in the sub-G1 population from 6.9% in untreated controls to 10.4% and 13.7% in cells treated with 10 and 20 μg/mL *Tc*CaNB, respectively, reinforces the conclusion that *Tc*CaNB triggers apoptosis in HeLa cells. This apoptotic response is consistent with the DNA fragmentation results previously discussed. The absence of significant changes in the cell cycle progression of Vero cells in the presence of *Tc*CaNB further supports the selective action of TcCaNB toward HeLa cells. The lack of observable alterations in the cell cycle of normal cells implies that *Tc*CaNB does not affect these cells' cell cycle dynamics in the same manner, aligning with the previously noted absence of apoptosis in Vero cells. This selectivity could be beneficial for minimizing off-target effects and reducing potential toxicity to normal tissues.

These findings have important implications for the development of *Tc*CaNB as a therapeutic agent. The selective induction of apoptosis in HeLa cells, coupled with minimal effects on normal cells, supports the potential use of *Tc*CaNB as a targeted anticancer drug. Further studies should investigate the exact molecular targets of *Tc*CaNB and explore its efficacy in other cancer cell lines and in vivo models to better understand its therapeutic potential and safety profile. The findings suggest that *Tc*CaNB exerts its cytotoxic effects through both cell cycle disruption and apoptosis induction. The inhibition of the S phase may contribute to the accumulation of DNA damage, leading to apoptosis. Understanding this dual mechanism is crucial to optimize the potential therapeutic use of TcCaNB and address potential resistance mechanisms.

Furthermore, the study of secreted calcium binding proteins in *T. cruzi* has gained importance, proteomic analysis of the secretome of *T. cruzi* revealed a rich content of proteins involved in metabolism, signaling, survival, and virulence of the parasite [56]. Among these proteins is found Calreticulin (*Tc*CRT) which is involved in host–parasite interaction, which exhibits antiangiogenic and antitumor properties in vitro and in vivo [31]. On the other hand, the study of the secretome has shown the presence of other calcium and calmodulin binding proteins, involved in cell signaling [56]. Research on recombinant proteins from *T. cruzi*, such as rP21, GP82, and *Tc*CRT, has opened new perspectives on the relationship between Chagas disease and cancer; here, it is important to consider the importance of the mechanisms associated with tumor protection mediated by different components of the parasite and their relationship with the inhibition of invasion, metastasis, and angiogenesis, highlighting the antitumor potential of these components. The ability of these proteins to directly impact key events in the cell cycle, apoptosis, or immune processes highlights their importance in the development of new therapeutic approaches in the field of oncology [57]. The study of recombinant proteins derived from *T. cruzi* also involves exploring the molecular mechanisms involved in their antitumor activity, potentially identifying specific therapeutic targets and opening avenues for the development of new cancer drugs. Similarly, the investigation of these proteins, including *Tc*CaNB, not only provides insights into their potential antitumor role but also contributes to understanding how the parasite could interact with and modulate host cells, which may have broader implications in the cellular and molecular biology of the parasite.

In summary, we demonstrate that *Tc*CaNB interacts with proteins on the surface of melanoma, cervical cancer adenocarcinoma, and renal epithelial cells, generating changes in the viability, proliferation, and cellular morphology of the tumor lines in vitro, without affecting the nontumor cell to a greater degree. In addition, *Tc*CaNB shows selective cytotoxic effects in HeLa cells by inducing apoptosis and disrupting the cell cycle, with minimal impact on normal cells. This supports its potential as a targeted anticancer drug. Further research is needed to identify its molecular targets and evaluate its efficacy in other cancer cell lines and in vivo models to better understand its therapeutic potential and safety profile. Understanding its dual mechanism of action, which includes cell cycle disruption and apoptosis, is crucial for optimizing its use and addressing potential resistance mechanisms. Finally, our findings provided new information on the antitumor effect of *Tc*CaNB, which is important for the further understanding and development of *Tc*CaNB as a new drug for cancer treatment.

## 5. Conclusion

This study represents the first comprehensive investigation of the extracellular role of *Tc*CaNB in tumor cell lines in vitro. We demonstrate that *Tc*CaNB interacts with cell surface proteins in melanoma (B16-F10), adenocarcinoma (HeLa), and renal epithelial (Vero) cells. Notably, *Tc*CaNB induces significant cytotoxic effects in tumor cells, including alterations in cell viability, proliferation, and morphology without affecting normal cells. The observed selective induction of apoptosis and cell cycle disruption in HeLa cells highlights *Tc*CaNB's potential as a targeted anticancer agent. The dual mechanism of *Tc*CaNB, involving both cell cycle arrest and apoptosis, underpins its efficacy in inducing tumor cell death. Further research is warranted to identify specific molecular targets of *Tc*CaNB. Understanding these mechanisms will be crucial for optimizing *Tc*CaNB's therapeutic application and overcoming potential resistance, thus advancing its development as a novel cancer treatment.

## Figures and Tables

**Table 1:** Quantification of cell cycle analyses obtained by flow cytometry on Hela cells treated with *Tc*CaNB.

HeLa cell population (%)				
Treatment	Sub-G1	G0/G1	S	G2/M
0 μg/mL *Tc*CaNB	6.9 ± 0.2	56.9 ± 1.1	13.8 ± 0.3	21.7 ± 0.9
5 μg/mL *Tc*CaNB	6.8 ± 0.8	56.1 ± 1.7	12.6 ± 1.1	23.1 ± 2.2
10 μg/mL *Tc*CaNB	10.4 ± 0.3	46.3 ± 1.0	17.4 ± 0.5	23.6 ± 0.5
20 μg/mL *Tc*CaNB	13.7 ± 1.1	33.7 ± 2.1	23.7 ± 3.8	24.1 ± 2.4

*Note:* Percentage of HeLa cell population distributed in the phases of the cell cycle.

**Table 2:** Quantification of cell cycle analyses obtained by flow cytometry on Vero cells treated with *Tc*CaNB.

Vero cell population (%)				
Treatment	Sub-G1	G0/G1	S	G2/M
0 μg/mL *Tc*CaNB	8.1 ± 1.4	48.6 ± 3.9	11.1 ± 2.3	19.5 ± 1.5
5 μg/mL *Tc*CaNB	8.3 ± 1.0	53.2 ± 2.9	10.6 ± 1.5	16.7 ± 4.2
10 μg/mL *Tc*CaNB	8.9 ± 0.1	52.0 ± 1.3	10.7 ± 2.1	16.2 ± 0.9
20 μg/mL *Tc*CaNB	9.9 ± 2.2	52.5 ± 2.8	9.7 ± 0.9	14.7 ± 2.4

*Note:* Percentage of Vero cell population distributed in the phases of the cell cycle.

## Data Availability

The data that support the findings of this study are available from the corresponding author upon reasonable request.

## Supporting Information

Supporting Information 1 Figure Supporting 1. Anti-*h/m/r* CaNB antibody evaluation for detection of *Tc*CaNB. 5 *μ*g of *Tc*CaNB protein was analyzed by Western blotting using the anti-*h/m/r*CaNB antibody. The obtained band is approximately 19 kDa. 10 *μ*g of rat BE was used as a positive control for CaNB.

Supporting Information 2 Figure Supporting 2. Far Western Blotting of recombinant *Tc*CaNB with membrane proteins from B16-F10, HeLa, and Vero cells. 100 *μ*g of membrane proteins from B16-F10 and HeLa cells and 30 *μ*g of recombinant *Tc*CaNB were used. The interaction was detected with anti-*Tc*CaNB and anti-*h/m/r* CANB antibodies. (A) Far WB using anti-*Tc*CaNB antibody for detection. (B) WB control using anti-*Tc*CaNB antibody. (C) Far WB using anti-*h/m/r* CaNB antibody. (D) WB control using anti-*h/m/r* CaNB antibody. (E) Far WB using anti-*h/m/r* CaNB antibody. (F) WB control using anti-*h/m/r* CaNB antibody. 10 *μ*g of BSA was used as negative control in Far WB. 100 *μ*g of B16-F10, HeLa, and Vero membrane proteins were used in WB controls.

Supporting Information 3 Figure Supporting 3. Half-maximal inhibitory concentration (IC50) of *Tc*CaNB. B16-F10, HeLa, and Vero cells were treated with different concentrations of *Tc*CaNB for 24 h. The IC50 values were determined using a dose–response curve.
