# Supplementary figures and images for: Cytotoxic Effect of Trypanosoma cruzi Calcineurin B Against Melanoma and Adenocarcinoma Cells In Vitro
Source: Adv Pharmacol Pharm Sci. 2024 Nov 28;2024:5394494. doi: 10.1155/adpp/5394494 (PMC11620811; doi:10.1155/adpp/5394494)

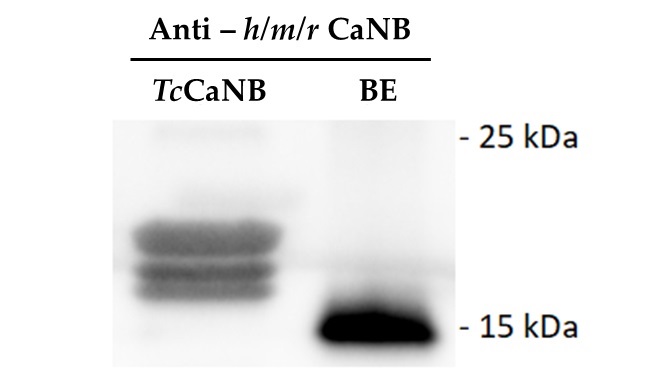

Supplement: Supporting Information 1 — Figure Supporting 1. Anti-h/m/r CaNB antibody evaluation for detection of TcCaNB. 5 μg of TcCaNB protein was analyzed by Western blotting using the anti-h/m/rCaNB antibody. The obtained band is approximately 19 kDa. 10 μg of rat BE was used as a positive control for CaNB. [file 5394494.f1.jpg]

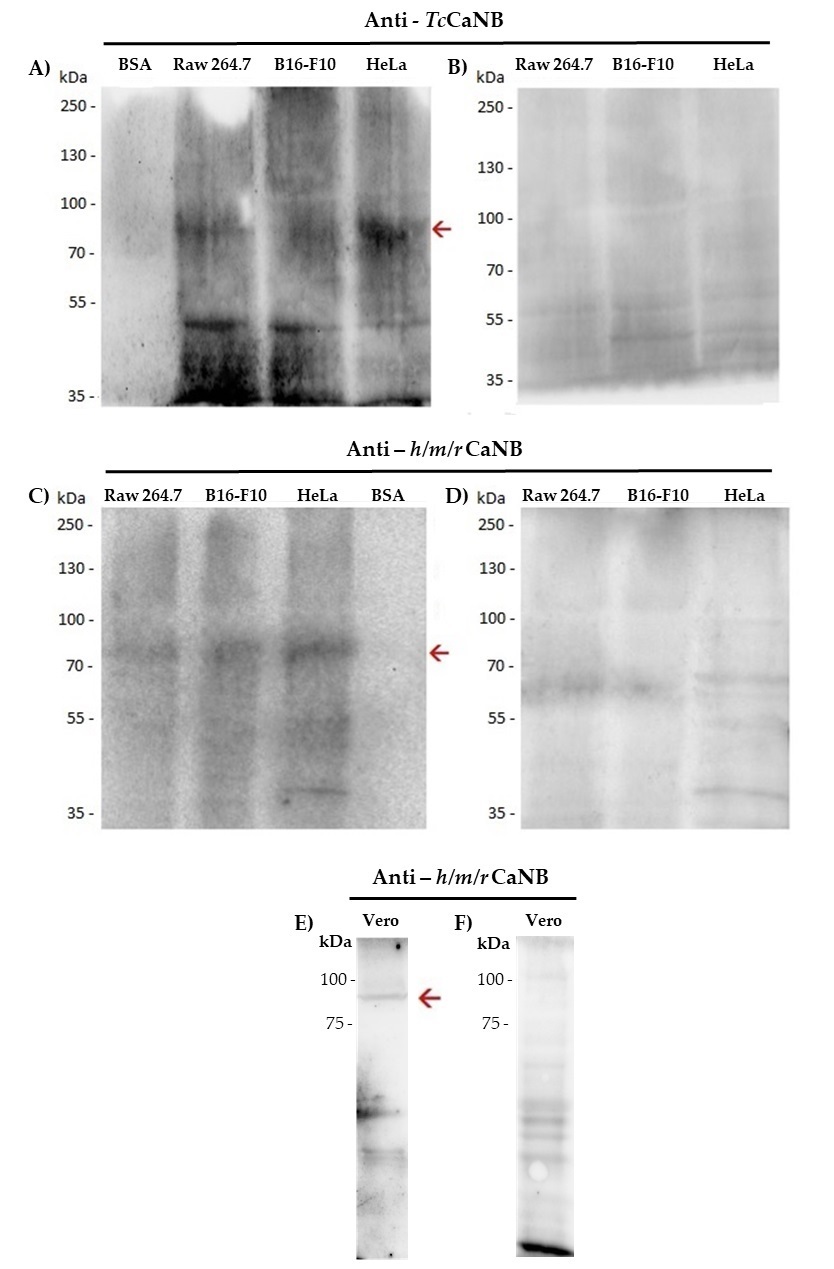

Supplement: Supporting Information 2 — Figure Supporting 2. Far Western Blotting of recombinant TcCaNB with membrane proteins from B16-F10, HeLa, and Vero cells. 100 μg of membrane proteins from B16-F10 and HeLa cells and 30 μg of recombinant TcCaNB were used. The interaction was detected with anti-TcCaNB and anti-h/m/r CANB antibodies. (A) Far WB using anti-TcCaNB antibody for detection. (B) WB control using anti-TcCaNB antibody. (C) Far WB using anti-h/m/r CaNB antibody. (D) WB control using anti-h/m/r CaNB antibody. (E) Far WB using anti-h/m/r CaNB antibody. (F) WB control using anti-h/m/r CaNB antibody. 10 μg of BSA was used as negative control in Far WB. 100 μg of B16-F10, HeLa, and Vero membrane proteins were used in WB controls. [file 5394494.f2.jpg]

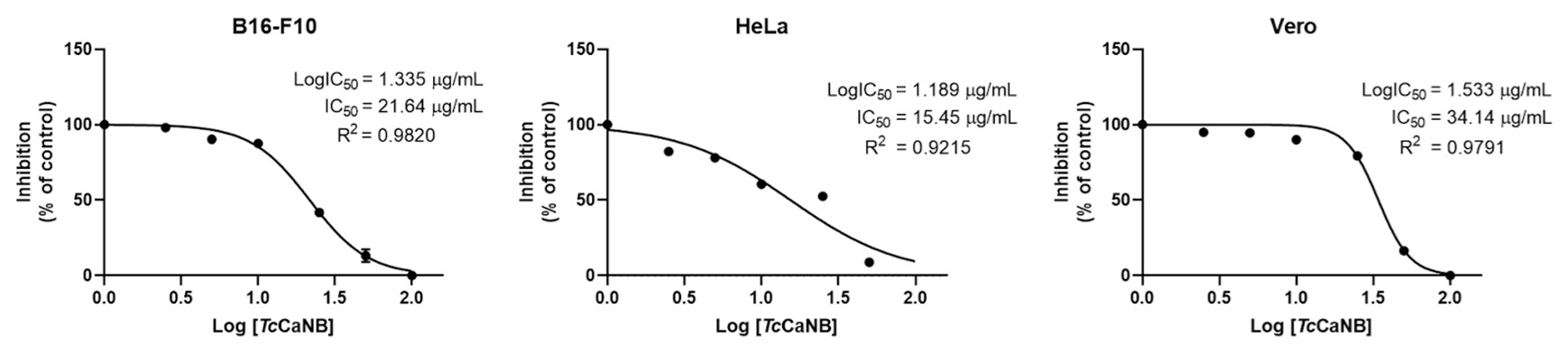

Supplement: Supporting Information 3 — Figure Supporting 3. Half-maximal inhibitory concentration (IC50) of TcCaNB. B16-F10, HeLa, and Vero cells were treated with different concentrations of TcCaNB for 24 h. The IC50 values were determined using a dose–response curve. [file 5394494.f3.jpg]
